# Phasing of muscle gene expression with fasting-induced recovery growth in Atlantic salmon

**DOI:** 10.1186/1742-9994-6-18

**Published:** 2009-08-24

**Authors:** Neil I Bower, Richard G Taylor, Ian A Johnston

**Affiliations:** 1 Gatty Marine Laboratory, School of Biology, University of St Andrews, St Andrews, Fife, KY16 8LB, UK; 2 EWOS Innovation, 4335 Dirdal, Norway

## Abstract

**Background:**

Many fish species experience long periods of fasting in nature often associated with seasonal reductions in water temperature and prey availability or spawning migrations. During periods of nutrient restriction, changes in metabolism occur to provide cellular energy via catabolic processes. Muscle is particularly affected by prolonged fasting as myofibrillar proteins act as a major energy source. To investigate the mechanisms of metabolic reorganisation with fasting and refeeding in a saltwater stage of Atlantic salmon (*Salmo salar L*.) we analysed the expression of genes involved in myogenesis, growth signalling, lipid biosynthesis and myofibrillar protein degradation and synthesis pathways using qPCR.

**Results:**

Hierarchical clustering of gene expression data revealed three clusters. The first cluster comprised genes involved in lipid metabolism and triacylglycerol synthesis (*ALDOB*, *DGAT1 *and *LPL*) which had peak expression 3-14d after refeeding. The second cluster comprised *ADIPOQ*, *MLC2*, *IGF-I *and *TALDO1*, with peak expression 14-32d after refeeding. Cluster III contained genes strongly down regulated as an initial response to feeding and included the ubiquitin ligases *MuRF1 *and *MAFbx*, myogenic regulatory factors and some metabolic genes.

**Conclusion:**

Early responses to refeeding in fasted salmon included the synthesis of triacylglycerols and activation of the adipogenic differentiation program. Inhibition of MuRF1 and MAFbx respectively may result in decreased degradation and concomitant increased production of myofibrillar proteins. Both of these processes preceded any increase in expression of myogenic regulatory factors and *IGF-I*. These responses could be a necessary strategy for an animal adapted to long periods of food deprivation whereby energy reserves are replenished prior to the resumption of myogenesis.

## Background

Fasting is a natural phenomenon in high latitude fish and is associated with low winter temperatures, short-days which restrict feeding opportunities and/or reduced availability of prey items. Atlantic salmon (*Salmo salar *L.) experience periods of fasting during the completion of their lifecycle, when adult fish return to freshwater to spawn. Reproduction and migration in anadromous salmonids requires substantial energy input, which is often mobilised from stores in visceral and somatic tissues [1]. These energy stores are mostly acquired in the ocean [2], since salmon cease feeding prior to and during their upstream migration to spawning grounds in freshwater [3,4]. Considerable energy is also used for gonadal development, nest construction, courtship and intrasexual competition [1,5-8]. Kelts (previously spawned adult salmon), which overwinter in fresh water pools and descend in spring [9], rely entirely on stored energy reserves to survive and are often in an emaciated condition [10]. Juvenile salmon may also experience fasting, as juveniles which show delayed levels of activity, delay their migration to the sea and overwinter in freshwater [11]. To survive the winter months, these fish rely heavily on lipid reserves accumulated during autumn [12-14].

Fasting in fish is associated with a reduction in metabolic rate at the whole animal level [15]. Reductions in protein synthesis with extended fasting are undoubtedly an important component of the observed metabolic depression [16]. Net changes in tissue mass are a function of the balance between protein synthesis and degradation. In rainbow trout liver prolonged starvation resulted in no change in fractional protein synthesis rates, a large decrease in absolute protein synthesis rates and increased protein degradation rates [17]. The amino acids produced from the net mobilisation of proteins from the myotomal muscles represent a major energy source for other tissues [18,19]. Fish myotomes contain slow and fast muscle fibre types primarily used in sustained and burst swimming respectively [20,21]. Biochemical and ultrastructural studies have shown that slow muscle is relatively spared from the effects of fasting, presumably because it is needed for continuous swimming at all speeds [22,23]. Atrophy in fast muscle follows depletion of glycogen and lipid stores and is associated with a decrease in myofibrillar diameter, and a preferential degradation of peripheral myofilaments [23]. Feeding fish to appetite following fasting or a period of growth restriction results in faster growth relative to continuously fed control groups, a phenomenon referred to as compensatory or catch-up growth [24,25]. The primary mechanism underlying compensatory growth with food restriction in Atlantic salmon is an increase in feeding intensity [25].

Fasting-refeeding protocols have become one of the main manipulative tools used to investigate the molecular and genetic mechanisms regulating growth [26-29]. As in mammals, Insulin-like growth factor-I (IGF-I) and IGF-II are the key hormones which stimulate protein synthesis in fish muscle [26,29,30]. IGF-I from the liver is delivered to the muscle by the circulation and in addition both IGF-I and IGF-II are locally synthesized in response to environmental and nutritional stimuli [31]. The complete IGF-system in fish comprises 4 splice variants, several membrane receptors and six IGF-binding proteins which act on the PI3K/AKT/mTOR pathway [31,29]. Rescan et al., [28] used a cDNA microarray containing 9023 rainbow trout (*Oncorhynchus mykiss*) sequences to provide a general description of some of the changes in muscle gene expression that accompanied recovery growth following fasting. Fasting was associated with an upregulation of cysteine protease cathepsins and components of the ubiquitin-proteosome involved in protein degradation as well as tuberous sclerosis component 2 (TSC2) an inhibitor of mTOR function and the translational repressor 4E-BP1 [28,32]. 4 to 11 days after refeeding there was a downregulation of genes involved in protein catabolism and an upregulation of genes involved in translation, protein folding and maturation and ribosome formation [28].

In mammals, the signals that regulate muscle atrophy and hypertrophy are linked through the PI3K/AKT/mTOR pathway [33]. Activation of the PI3K/AKT/mTOR pathway by IGF-I causes a phosphorylation cascade that leads to an increase in translation and therefore protein synthesis, resulting in skeletal muscle hypertrophy [34]. Phosphorylation of AKT also results in inhibition of key regulators of skeletal muscle atrophy, the muscle specific ubiquitin ligases MAFbx and MuRF1, through phosphorylation of the FOXO transcription factors [35]. Conversely, dephosphorylation of FOXO transcription factors, for example during nutrient restriction, results in increased expression of MuRF1 and MAFbx. MAFbx and MuRF1 are E3 ubiquitin ligases involved in the targeting of proteins, such as myosin light chain 2, for degradation via the ubiquitin pathway [35,36]. MAFbx targets elongation initiation factor 3-f (eif3-f) for degradation, a key regulator of mTOR mediated translation of muscle structural proteins including myosin heavy chain and desmin [37,38]. MuRF1 has also recently been shown to depress energy metabolism in mammalian muscle via effects on pyruvate dehydrogenase and creatine kinase [39]. Furthermore, MyoD, a master transcriptional factor for myogenesis is a target of MAFbx [40], indicating a widespread role of ubiquitin ligases in regulating muscle growth in mammals.

Quantitative PCR (qPCR), if performed with appropriate normalisation, statistical analysis and under standardised operating procedures such as those described in the Minimum Information for publication of Quantitative real-time Experiments guidelines (MIQE), is the method of choice for reliably quantifying changes in gene expression [41]. In the present study, qPCR was used to investigate the phasing of gene expression in the fast muscle of Atlantic salmon during fasting-induced recovery growth. Since the response of both *MuRF1 *and *MAFbx *expression to fasting-refeeding has not been investigated in Atlantic salmon we particularly wanted to determine the expression of *MuRF1 *and *MAFbx *in relation to *IGF-I *and marker genes for myogenesis, glucose homeostasis and lipid metabolism.

## Methods

The methods described in this paper for qPCR analysis of gene expression are compatible with the MIQE guidelines [41].

### Fish and experimental design

All experiments were conducted at EWOS Innovation, Dirdal, Norway and were approved by the local animal welfare committee. Atlantic salmon (*Salmo salar. L *1327 g ± 336.1 g, Mean ± SD, n = 55) were individually passive induced transponder (PIT) tagged (Fish Eagle, Lechlade, Gloucestershire England) so that growth rate could be calculated. Fish were fasted for 32 days then fed to satiation with a commercial feed (EWOS Innovation, 52% fish meal, 13% rape seed oil, 12% wheat protein, 10% fish oil, 5% pea protein, 5% soy protein, 3% krill meal, vitamin C-35 0.29%, vitamin mix 0.15%, vitamin E-50 0.03%, methionine 0.02%) for 32 days. The average daily temperature was 7.8°C, oxygen was 13.96 mg L^-1^, and average daily salinity was 28.9 ppm. Samples were taken at 0 d, 3 d, 7 d, 14 d and 32 d following refeeding with 8 fish sampled at each time point. Fish were humanely killed by Schedule 1 of the Animals (Scientific Procedures) Act 1986 (Home Office Code of Practice. HMSO: London Jan. 1997) and individual mass and fork lengths measured. Fast muscle was dissected from the dorsal myotome between 0.6 and 0.7 standard length (tip of snout to last vertebrae). Tissues were snap frozen in liquid nitrogen and stored at -80°C until analysed. Growth was measured as the Thermal Growth Co-efficient (TGC = [(M_2_^0.333^-M^0.333^)(degree days)^-1^*1000] where M_1 _and M_2 _were start and final body weights respectively. Degree days values are the sum of the °C values for each day of the experiment.

### RNA extraction and cDNA synthesis

Total RNA was extracted by addition of 100 mg of muscle to Lysing matrix D (Qbiogene, Irvine, California) with 1 ml Tri Reagent (Sigma, Gillingham, Dorset, UK) and homogenised using a Fast Prep instrument (Qbiogene, Irvine, California). Total RNA was quantified based on absorbance at 260 nm. Only samples with a A260/280 ratio between 1.8 and 2.1, and a an A260/230 ratio above 1.8 were used for reverse transcription. Genomic DNA contamination was removed by treatment with Turbo DNA-free (Ambion, Austin, Texas, USA), and the integrity of purified RNA confirmed by agarose gel electrophoresis. First strand cDNA was synthesised from 1 ug total RNA using Superscript III (Invitrogen, Carlsbad, CA, USA) as per manufacturer's guidelines

### Primer design

Previously published primers were used for LPL [42]. Primers were designed using Net primer (Premier BioSoft) to have Tm of 60°C, and where possible, were designed to cross an exon-exon junction to avoid amplification of contaminating genomic DNA. The primers, amplicon size, amplicon melting temperature and accession numbers of genes used for qPCR are listed in table 1. The following genes were studied: *Insulin-like growth factor I *(*IGF-I*), *myogenin *(*MYOG*), *myoblast determination factor 1 *(*myoD1a*), *myocyte enhancer factor 2A *(*MEF2A*), *muscle ring finger protein 1 *(*MuRF1*), *muscle-specific X box protein *(*MAFbx*), *myosin heavy chain *(*MHC*), *myosin light chain 2 *(*MLC2*), *pyruvate kinase *(*PKM*), *phosphoglycerate kinase *(*PGK*), *transaldolase *(*TALDO1*), fructose *1,6 bisphosphatase *(*FBP1*), *Aldolase B *(*ALDOB*), *cyclic AMP response element binding protein *(*CrebA*), *lipoprotein lipase *(*LPL*), *diacylglycerol O-acyltransferase homolog 1 *(*DGAT1*), and *adiponectin *(*ADIPOQ*).

**Table 1:** Summary of parameters for qPCR genes studied.

Gene	Primer 5'-3'	Prod. size (bp)	Tm (°C)	E (%)	R^2^	Accession number
IGF-I	f:CCTGTTCGCTAAATCTCACTTC	226	80.3	100.3	0.998	EF432852
	r:TACAGCACATCGCACTCTTGA					
Myogenin	f:GTGGAGATCCTGAGGAGTGC	146	84.5	95	0.993	DQ294029
	r:CTCACTCGACGACGAGACC					
MyoD1a	f;CCAAATAGTTCCAGACGCAAG	104	79.8	102.1	0.999	AJ557148
	r:ACAGCGGGACAGGCAGAGG					
MEF2A	f:ACCGGCTACAACACCGAGTA	121	84.1	92.5	0.994	DY713536
	r:CCTGGCCCAGTTGATGTT					
MuRF1	f:AGGCGGGATCAGAGCTAAC	229	87.2	103.7	0.998	DN165465
	r:CGACCATTCCAAAGTCCATC					
MAFbx	f:AAAGGAAGCACTAAAGAGCGTC	137	83.6	97.2	0.996	DN165813
	r:CTGGGACTTGGCAATGAGC					
Pkm	f:GTGACCATGATGCACTCGATC	225	84.6	100.3	0.992	CK888371
	r:GGACAGCGTGGGCGATAC					
Pgk	f:CTCGGTGATGGGGCTTAGG	160	82.6	92.1	0.996	DN166327
	r:TCATTGGTGGAGGCGACA					
TALDO1	f:AGGTAGACGCCAGGCTTTC	125	82.4	99	0.994	EG912503
	r:CCATGTTGAGGAGAGCTTGA					
FBP1	f:TGGGATTGCCAACCTCTATG	153	81.3	96.9	0.996	EG896159
	r:GCCCTCTCGTTCTCCTCTG					
ALDOB	f:TCCGTGACCTCCTGTTCTCT	159	83.5	102.1	0.998	AF067796
	r:CTGTGCCTTTGTCCACCTTA					
CrebA	f:GGAGTCTGTTTCGCTAAGTCG	168	84.1	100.1	0.998	CU073780
	r:CGTAGGACCGCTGGATGT					
LPL	f:TGCTGGTAGCGGAGAAAGACAT	114	80.8	95.9	0.998	BI468076
	r:CTGACCACCAGGAAGACACCAT					
ADIPOQ	f:CCAGCCAGAAGGCAATGTAT	192	81.2	98.6	0.996	EG776984
	r:CACCAACGACTCCACCTTC					
MHC	f:GCACGCCACTGAAAAC	209	84.1	94.2	0.996	DN164736
	r:CCTCAAGGTCGTCCACT					
DGAT1	f:CATGCTGGAGGTGATG	222	80.1	96.5	0.998	DW564359
	r:GGAAGCACAGTGTGACTGA					
MLC2	f:TCAACTTCACCGTCTTCCTCAC	194	82.6	98.5	0.994	NM_001123716
	r:GCCCACAGGTTCTTCATCTCC					
EF1-α	fATCGGCTATGCCTGGTGAC	141	85	96.3	0.999	BG933853
	r:ATGATGACCTGAGCGGTG					
B-actin	fACCCAGATCATGTTTGAGACC	146	82.9	92.7	0.997	G933897
	r:TCGTAGATGGGTACTGTGTGGG					
RNA pol II	f:TACATGACCAAATATGAAAGG	157	84.5	94.6	0.998	BG936649
	r:GATGATGGGGATCTTCCTGC					
HPRT1	f:CCTCAAGAGCTACTGTAAT	255	80.8	93.6	0.997	EG866745
	r:TCTGGAACCTCAAACCCTATG					
18S	f:GCGTCCAACTTCTTA	189	85.7	95.3	0.998	AJ427629
	r:CAATCCCCAATCCCTATC					

Forward and reverse primer sequences (5'-3'), amplicon product size in base pairs (bp), melting temperature of amplicon (Tm), PCR efficiency (E), regression analysis of plasmid dilution series (R^2^) and accession numbers for genes used in qPCR.

### Quantitative PCR

qPCR was performed using a Stratagene MX3005P QPCR system (Stratagene, La Jolla, CA, USA) with SYBR green chemistry (Power SYBR, Applied Biosystems, Foster City, CA, USA). cDNA used in qPCR assays was first diluted 80-fold with nuclease free water. Each qPCR reaction mixture contained 7.5 μl 1 × Power SYBR green master mix, 6 μl cDNA (80-fold dilution), 500 nM each primer and RNase free water to a final volume of 15 μl. Amplification was performed duplicate in 96 well plates (Stratagene, La Jolla, CA, USA) with the following thermal cycling conditions: initial activation 95°C 10 minutes, followed by 40 cycles of 15 s at 95°C, 30 s at 60°C, and 30 s at 72°C. Dissociation analysis of the PCR products was performed by running a gradient from 60 to 95°C to confirm the presence of a single PCR product. Products were also sequenced to confirm identity. A dilution series made from known concentrations of plasmid containing the PCR inserts was used to calculate absolute copy numbers for each of the genes examined.

Standards for calculating absolute copy number for each gene were prepared by cloning the PCR product from each primer pair into a T/A pCR4-TOPO vector (Invitrogen, Carlsbad, CA, USA) and transformation of chemically competent TOP10 *Escherichia coli *cells (Invitrogen,. Carlsbad, CA, USA). Individual colonies were grown and plasmids purified using Fastprep plasmid purification method (Eppendorf, Hamburg, Germany). The concentration of each plasmid was calculated based on absorbance at 260 nm, and a four fold dilution series produced for calculation of copy number via qPCR. The r^2 ^values and qPCR efficiencies based on the plasmid standards dilution series are given in table 1.

### Data analysis

Genorm [43] was used to analyse the stability of several reference genes including *18S Ribosomal RNA*, *hypoxanthine phosphoribosyltransferase 1 *(*HPRT1*), *β-actin*, *RNA polymerase II *and *Elongation factor 1-α *(*EF1-α*). Analysis revealed *HPRT1*, *RNA polymerase II *and *β-actin *to be the most stable genes in this experimental system (M = 0.41), thus the geometric average of these genes was used for normalisation of qPCR data. Statistical analysis was performed using minitab (Minitab Inc). Significant differences in expression between time points were calculated by ANOVA using Fisher's individual error rate post hoc tests. Correlations in gene expression were calculated using linear regression and Pearson's correlation. Hierarchical clustering was performed using Cluster3 software [44].

## Results

### Fish Growth characteristics

**Figure 1:**
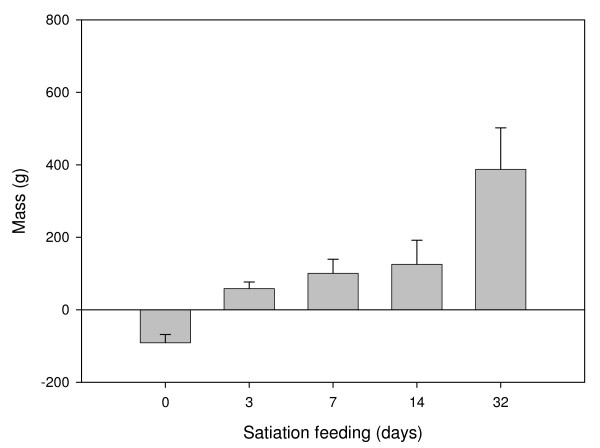
**Change in body mass over the time course of the experiment**. Fish were fasted for 32 days, and then fed to satiation with a commercial fish feed (EWOS Innovation). Values represent mean ± S.D., n = 8 per sample point. The mass of fish at day 0 was 1327 g ± 336.1 g, (Mean ± SD, n = 55)

Prior to fasting, fish had an average mass of 1327 g ± 336.1 g (Mean ± SD, n = 55), which after fasting for 32 days, had reduced by an average of 91.2 ± 23.1 g (Fig. 1), with an average TGC of -1.0 ± 0.14. After feeding, weight gains were 58.4 ± 18.3 g, 100.6 ± 38.7 g, 125.4 ± 66.6 g and 387.6 ± 114.3 g for days 3, 7, 14 and 32 respectively (n = 10). Early TGC calculations are unreliable as the food present in the gut gives a false indication of growth whereas the later time points provide a more reliable estimate. The TGC for fish (when calculated from weight at the start of feeding, day 0) was mean 3.1 ± 1.1, n = 8 at 14 d and mean 3.7 ± 0.8, n = 8 at 32 d. At day 3, there was an initial increase in weight of 58.4 ± 18.3 g, contributed by the presence of food in the gut. If this value is subtracted from the later time points, then actual weight gain for days 7, 14 and 32 are 42.2 ± 24.9 g, 67.0 ± 48.1 g and 329.2 ± 114.2 g.

### Gene expression

#### Overview

**Figure 2:**
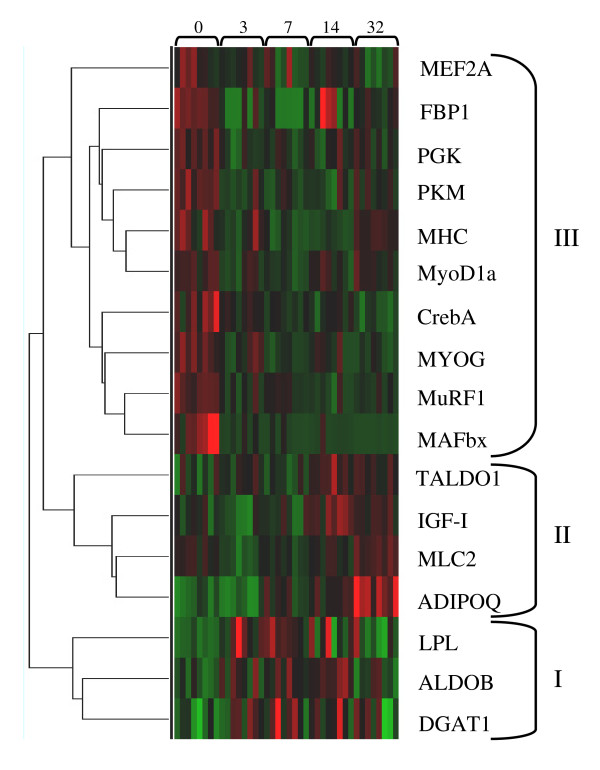
**Heat map summary and hierarchical clustering of gene expression patterns during refeeding of fasted (day 0) Atlantic salmon from 3 to 32 days**. Spearman rank correlation method using the Cluster3 program was used for hierarchical clustering. Rows are standardised to have a mean of 0 and s.d of 1, so that red indicates high, green low and black values equal to zero. Based on expression patterns, 4 gene clusters were identified. Cluster I comprised genes which had peak expression at 3-14d after refeeding. Cluster II comprised genes showing peak expression 14-32d after refeeding. Cluster III contained genes which had high expression in fasted fish and mostly down regulation throughout refeeding.

A summary heat map and hierarchical clustering of gene expression patterns during fasting-induced recovery growth is shown in Fig. 2. Three main clusters of gene expression were identified. Cluster I comprised *ALDOB*, *DGAT1 *and *LPL *which had peak expression at 3-14d after refeeding (Fig. 2). Cluster II comprised *ADIPOQ*, *MLC2*, *IGF-I *and *TALDO1 *which were later responding showing peak expression 14-32d after refeeding (Fig. 2). In this cluster MLC2 also showed high transcript levels in fasted fish (Fig. 2). Cluster III contained the largest number of genes (*MuRF1, MAFbx, CrebA, MYOG, MEF2A*, *FBP1*, *PGK*, *PKM*, *MHC *and *MyoD1a*) which were mostly up-regulated in fasted fish and generally down regulated with refeeding (Fig. 2).

#### Muscle-specific ubiquitin ligases

**Figure 3:**
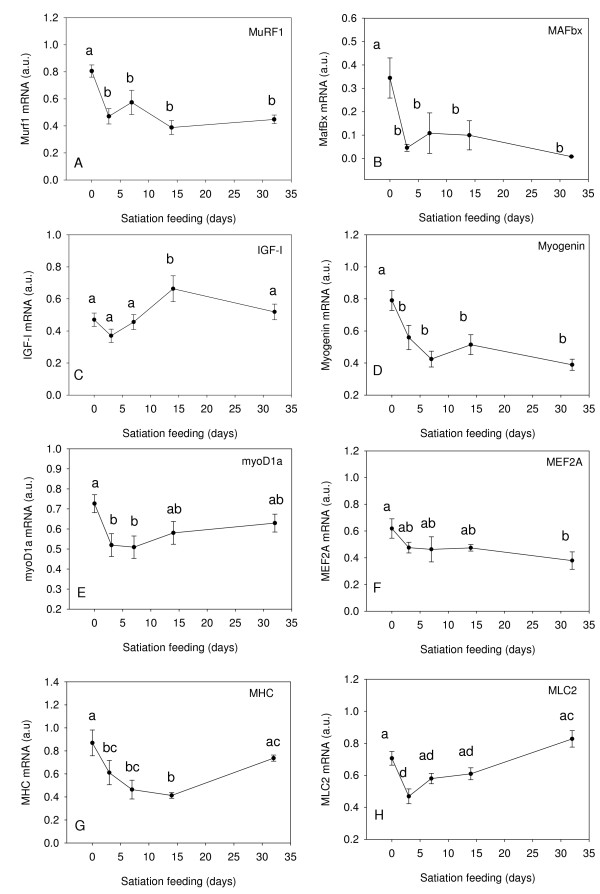
**Expression profiles for *MuRF1*(A), *MAFbx *(B), *IGF-I *(C), *MYOG *(D),*myoD1a *(E), *MEF2A *(F), *MHC *(G) and *MLC2 *(H) in fast muscle of Atlantic salmon from fasted fish (0 d), fed to satiation at 3 d, 7 d, 14 d, 32 d**. Gene expression was normalised to the geometric average of three reference genes (Genorm analysis), see text for details. Values represent mean ± S.E., 8 fish per sample point. Significant differences between means are indicated by different letters.

Both the muscle specific E3 ubiquitin ligases, *MuRF1 *and *MAFbx *were significantly down regulated at all time points in fed relative to the fasted fish (P < 0.01, Fig. 3A, B). *MAFbx *transcript levels were down regulated up to 98% (Fig. 3B)

#### IGF-I

Transcripts of *IGF-I *in fast muscle were similar in fasted fish and 3 d, 7 d and 32 d after feeding, but were significantly upregulated by 142% at 14 d (P < 0.05) compared to the 0 d sample (Fig. 3C)

#### Myogenic regulatory factors

The myoD family of transcription factors regulate the commitment of myoblasts to the myogenic lineage (myoD) and muscle differentiation (MYOG) [45]. *MYOG *expression decreased significantly between the 0 and 7d samples (P < 0.01) and then remained relatively constant with refeeding (Fig. 3D). There are three retained paralogues of myoD1 in Atlantic salmon each with distinct expression patterns during development and in different fibre types [46]. *MyoD1a *is the paralogue predominantly expressed in fast skeletal muscle [47]. *MyoD1a *was upregulated with fasting and showed only minor differences between the fed samples (Fig. 3E). The myocyte-enhancer 2 gene family (MEF2) proteins act co-operatively with myoD proteins to regulate myogenesis [48]. *MEF2A *transcripts were upregulated in starved individuals and tended to decrease with feeding, producing a statistically significant difference after 32 d (p < 0.05, Fig. 3F).

#### Myofibrillar proteins

The primers to myosin heavy chain were designed to conserved regions of the protein and could potentially amplify multiple isoforms of the *MHC *transcripts in fast muscle. Expression of this gene(s) was relatively high in fasted fish and significantly decreased 0-14d (P < 0.05) after feeding before increasing to 84% of fasted levels after 32 d (Fig 3G). Myosin light chain 2 significantly decreased (P < 0.01) an average 66% between fasted fish and the 3 d feeding sample before increasing to reach 117% of day 0 levels in the 32 d sample (Fig. 3H), although this increase was not statistically significant.

#### Metabolic genes

**Figure 4:**
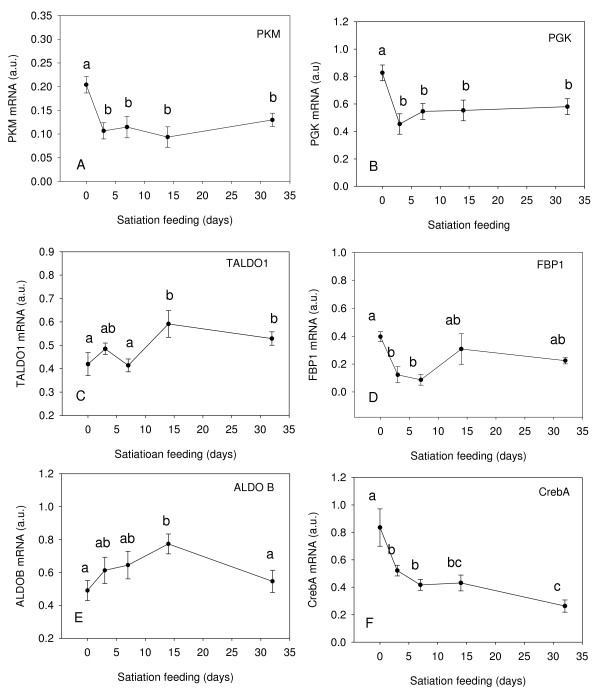
**Expression profiles for *PKM *(A), *PGK *(B), *TALDO1 *(C), *FBP1 *(D), *ALDOB *(E) and *CrebA *(F) in fast muscle of Atlantic salmon from fasted fish (0 d), fed to satiation at 3 d, 7 d, 14 d, 32 d**. Gene expression was normalised to the geometric average of three reference genes (Genorm analysis), see text for details. Values represent mean ± S.E., 8 fish per sample point. Significant differences between means are indicated by different letters.

**Figure 5:**
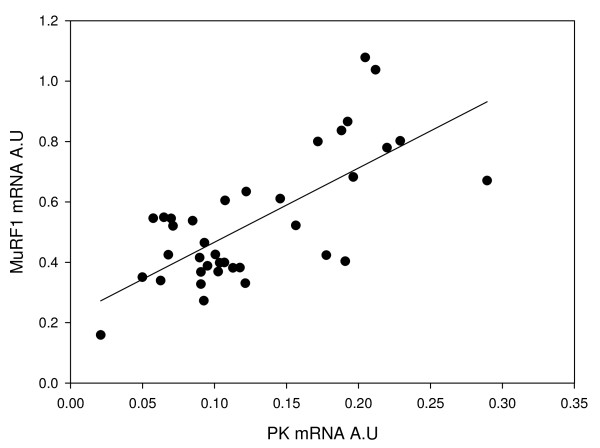
**Correlation between *MuRF1 *and *PKM *mRNA (r^2 ^= 0.50, p < 0.0001, n = 32)**.

Transcripts for *PKM *(Fig. 4A) and *PGK *(Fig. 4B) decreased by 50% between the fasted and 3 d sample (p < 0.05) and showed no further change with feeding. In contrast, levels of *TALDO1 *transcripts were similar in the fasted, 3 d and 7 d samples before increasing 141% at 14 d and 32 d (p < 0.05) (Fig. 4C). *FBP1 *mRNA was down regulated 69% at 3 d and 78% at 7 d (p < 0.05) relative to the fasted sample before partially recovering 14d and 32 d after refeeding (Fig. 4D). *ALDOB *transcripts increased with feeding reaching a peak at 14d which was significantly higher than in fasted fish (P = 0.05) (Fig. 4E). *CrebA *was down regulated 38% between the fasted and 3 d fed sample (p < 0.05) and showed a further 69% decline by the 32d sample (Fig. 4F). PKM expression was positively correlated with expression of MuRF1 (r^2 ^= 0.69, P < 0.0001, r = 0.71; Figure 5).

#### Genes involved in lipid metabolism and adipocyte differentiation

**Figure 6:**
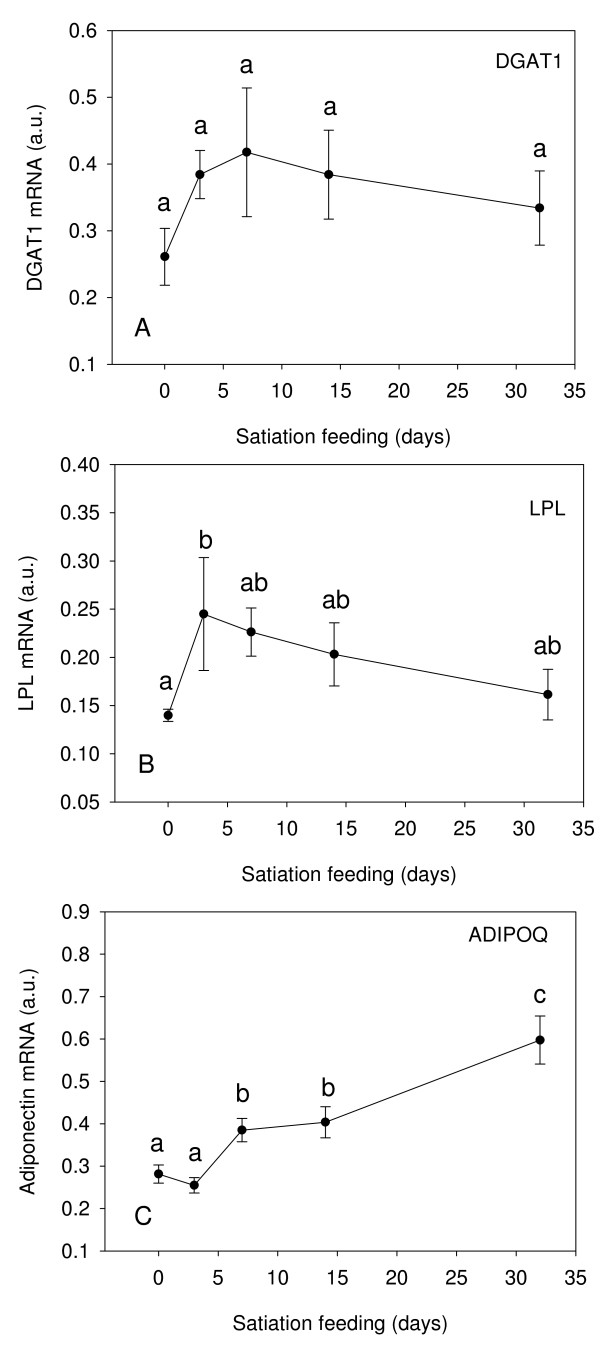
**Expression profiles for *DGAT1 *(A), *LPL *(B) and *ADIPOQ *(C) in fast muscle of Atlantic salmon from fasted fish (0 d), fed to satiation at 3 d, 7 d, 14 d, 32 d**. Gene expression was normalised to the geometric average of three reference genes (Genorm analysis), see text for details. Values represent mean ± S.E., 8 fish per sample point. Significant differences between means are indicated by different letters.

**Figure 7:**
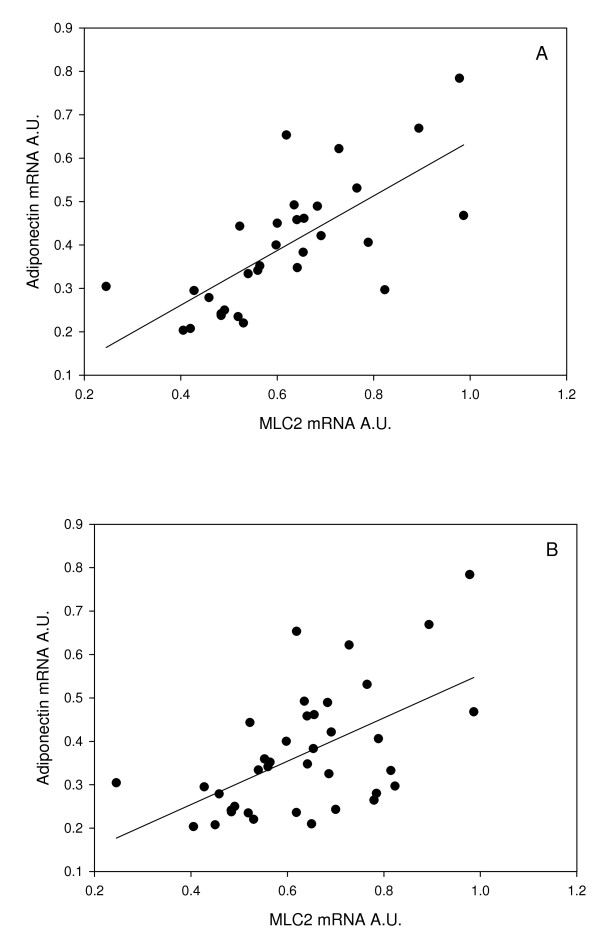
**Correlation between *MLC2 *mRNA and *ADIPOQ *mRNA at all time points (A) (r^2 ^= 0.36, p = 0.003, r = 0.59, n = 40), and at fed time points (B) (3 d, 7 d 14 d, 32 d; r^2 ^= 0.48, p < 0.001, r = 0.69 n = 32)**.

Expression of *DGAT1 *showed a small increase over fasting levels after 3 d of feeding and remained at this level throughout the experiment, although this increase was not statistically significant (Fig. 6A). Expression of *LPL *was significantly upregulated at 3 d, expression then decreased at 7 d and 14 d and returned to near day 0 values at 32 d (Fig. 6B). *ADIPOQ *transcript levels were significantly increased at 7 d, 14 d and 32 d compared to fasted fish (Fig. 6C). *ADIPOQ *was positively correlated with expression of MLC2 at all time points (r^2 ^= 0.35 p = 0.003, r = 0.60, Fig. 7A) and even higher correlation at fed time points (3 d, 7 d, 14 d, 32 d) (r^2 ^= 0.48 p < 0.0001, r = 0.69, Fig. 7B)

## Discussion

Atlantic salmon continue to grow throughout their life cycle. It is known that muscle fibres are continuously produced until ~2 kg body mass, after which growth only involves fibre hypertrophy and associated nuclear accretion [49]. Thus salmon of the size studied show active myogenesis. A 32d fast at 8°C, resulted in negative growth (TGC = -1.0) corresponding to a 6.9% decrease in body mass (Fig. 1). Following satiation refeeding, there was an average weight gain of 5.4% and 26.7% after 14d and 32d respectively. The myofibrillar proteins, principally actin and myosin, are a major source of the amino acids mobilised during fasting and migration in salmonids and comprise two thirds of the total protein content of fast muscle [50]. In the present study, mRNA transcripts for *MHC *and *MLC2 *decreased as an early response to feeding as reported earlier for Atlantic salmon undergoing a transition from zero to fast growth [29]. Expression of both these genes increased markedly by 32 d refeeding, with *MLC2 *expression exceeding that at 0 d (Fig. 3H).

Mammals respond to muscle wasting conditions, such as starvation, with the transcriptional upregulation of the ubiquitin ligases MuRF1 and MAFbx [51]. These proteins function in the ubiquitinisation of contractile proteins including troponin I [52] and myosin [53,54]. Furthermore, decreased MAFbx leads to an increase in the translation regulator eif3-f [38]. In the present study, we found that expression of the E3 ubiquitin ligases *MAFbx *and *MuRF1 *were also elevated in fasted Atlantic salmon and that both were significantly and strongly down regulated in response to feeding (Fig. 3A, B). *MAFbx *formed a cluster with *MuRF1*, *MYOG *and *CrebA*, which had strong down regulation at all time points following refeeding and with *myod1a*, *MHC*, *PKM, PGK, FBP1 and MEF2A*, which were also down regulated but not as strongly, following refeeding (Fig. 2). A reduction in protein degradation through down regulation of *MuRF1*, together with a concomitant increase in translational efficiency mediated by *MAFbx *may well allow the replacement of myosin mobilised during fasting much earlier than indicated by changes in transcript levels.

During fasting in mammals, glucagon release causes an increase in cAMP levels, activating the transcription factor CrebA [55], which induces the expression of genes involved in gluconeogenesis [56]. Due to the presence of cyclic AMP response elements in the promoter of CrebA, expression of CrebA is induced in a positive feedback mechanism [57]. In the present study, expression of *CrebA *was significantly down regulated in response to feeding as was *FBP1 *consistent with relatively high levels of gluconeogenesis occurring with fasting and a reduction in gluconeogenic activity with feeding.

In mammals, MuRF1 has been shown to decrease the abundance of certain glycolytic enzymes [39]. Thus in fasted fish elevated levels of MuRF1 might be expected to lead to a reduction in the flux through the glycolytic pathway. However, we found that the mRNAs for *PKM *and *PGK *were highest in fasted fish and decreased with feeding (Fig. 4). Interestingly, expression of *PKM *and *MuRF1 *were correlated (r^2 ^= 0.48, P < 0.0001, r = 0.71; Fig 5). It is possible that the elevated levels of *PKM *and *PGK *transcripts in fasted fish represent a response to decreased flux through the glycolytic pathway due to MuRF1 inhibition. Decreased flux through the glycolytic pathway could result in elevated levels of glucose which is a potent stimulator of *PKM *expression [58]. Johansen and Overturf [59] also found that expression of *PKM *increased during starvation and suggested that this increase was a requirement for the catabolisation of certain amino acids.

An alternative fate for the increased glucose present with feeding is to enter the pentose phosphate pathway [60]. TALDO1 is a key regulator of the pentose phosphate pathway, responsible for the generation of NADPH required for fatty acid synthesis, and ribose-5-phosphate required for nucleotide and nucleic acid synthesis [61,62]. Increased expression of TALDO1 is indicative of activation of the pentose phosphate pathway. Increased flux through this pathway could provide ribose-5-phosphate required for nucleotide production during the period of rapid growth with the NADPH used for fatty acid synthesis to replace the fatty acids used during the period of nutrient restriction. Generation of NADPH is also required to maintain glutathione in a reduced state and protect cellular integrity from reactive oxygen species [63]. The production of reactive oxygen species has been demonstrated to be lower in caloric restricted rats [64]. Increased expression of transaldolase may be required to protect cells from the elevated production of reactive oxygen species with refeeding.

In salmonids, muscle is a major site for fat storage [65]. In Atlantic salmon fast muscle, the fat content makes up 9.6% of the wet weight, with triacylglycerols contributing 93.3% of the total lipid content [66]. These reserves are likely to be exhausted during fasting, and must be replenished during periods of feeding. *ALDOB *is involved in the synthesis of triacylglycerols via the phosphatidic acid pathway [67]. Johanssen and Overturf [59] found that levels of *ALDOB *were 10 times higher during refeeding than in normal feeding, implying that during fasting-induced recovery growth, higher rates of fatty acid deposition are occurring. Also, Witt et al. [36] identified aldolase as a target for MuRF1, so the elevated levels of MuRF1 could further lead to reduced levels of aldolase in fasted fish.

Analysis of gene expression patterns reveals a cluster of genes (cluster I, Fig. 2) corresponding to triacylglycerol synthesis and adipogenic differentiation, which increase prior to any increases in MRFs or IGF-I (Fig. 2). LPL hydrolyses triglycerides, with the free fatty acids produced available as a direct energy source or used for storage in adipocytes [68]. Together, with the increased aldolase expression, increased LPL expression in the first few days of feeding, suggests TAGs are resynthesised in skeletal muscle to replace those used during the period of nutrient restriction. In Atlantic salmon, high densities of adipocytes are present in the myosepta of white muscle where the majority of lipids are stored as triacylglycerols [69]. We also examined the expression of *adiponectin *(*ADIPOQ*), an adipocytokine which increases in expression during differentiation of adipocytes and thus may serve as a marker of adipocyte differentiation [70,71]. Expression of this gene was significantly upregulated from 7 d after refeeding, indicating that adipogenic differentiation is occurring during these times. The expression of *Diacylglycerol:acyl CoA acyltransferase *(*DGAT1*), an enzyme involved in TAG synthesis, was also examined. Expression of *DGAT1 *increased early in response to feeding and remained elevated. *DGAT1 *mRNA expression has been shown to increase in differentiating 3T3-L1 adipocytes, with even greater increases in protein activity observed, suggesting that DGAT1 is also post transcriptionally regulated [72]. Although *LPL *and *ALDOB *gene expression decreased at day 32, *DGAT1 *remained elevated indicating that TAG synthesis is still occurring.

The decreased expression *ALDOB *and *LPL *after 32 d refeeding coincides with the increased expression of *MLC2 *and *MHC *(relative to 3 d, 7 d and 14 d). We found a positive correlation between *MLC2 *and *ADIPOQ *(r^2 ^= 0.35, P < 0.0005, r = 0.59; Fig. 7A), which was even greater when only days where food has been ingested are considered (r^2 ^= 0.48, P < 0.0001, r = 0.69; Fig. 7B). ADIPOQ has been demonstrated to increase sensitivity of myogenic cells to insulin [73]. The mTOR signalling pathway, which regulates protein accretion in skeletal muscle, is regulated by amino acids and insulin in teleost fish [74] so this increased sensitivity to insulin may be necessary for the resumption of myogenesis. Recently, cross talk of signals between skeletal muscle and adipose tissues has been suggested, with the cytokines identified as potentially important regulators maintaining the ratio of skeletal muscle to adipose tissue [75]. Receptors for ADIPOQ have been found to be expressed in zebrafish muscle [76]. Furthermore, myostatin has been shown to inhibit myogenesis and promote adipogenesis in multipotent mesenchymal cells [77]. Additional experiments in Atlantic salmon are required to determine if crosstalk between adipocytes and skeletal muscle is occurring and if this plays any role in regulating the myogenic program following refeeding.

## Conclusion

In conclusion, after a period of fasting, refeeding Atlantic salmon results in changes in metabolism leading to the replacement of lost energy reserves through increased fatty acid deposition and replacement of myofibrillar proteins. Increased myofibrillar protein deposition likely occurs through the down-regulation of *MuRF1 *and *MAFbx *leading to decreased protein degradation and increased translation respectively. Both the replacement of myofibrillar proteins and activation of the adipogenic program precede an increase in transcripts for myofibrillar proteins and myogenic regulatory factors. For an animal which is adapted to long periods of food deprivation, such as Atlantic salmon, achieving a state where energy reserves have been replenished, before metabolic energy is directed towards production of new muscle fibres, could be a necessary strategy for long term survival.

## Competing interests

The authors declare that they have no competing interests.

## Authors' contributions

NB performed the experimental work and wrote the first draft of the manuscript. RT was responsible for fish maintenance. IJ contributed to study design and writing of the manuscript. All authors read and approved the final manuscript
